# Nanohybrid of Co_3_O_4_ Nanoparticles and Polyphosphazene-Decorated Ultra-Thin Boron Nitride Nanosheets for Simultaneous Enhancement in Fire Safety and Smoke Suppression of Thermoplastic Polyurethane

**DOI:** 10.3390/polym14204341

**Published:** 2022-10-15

**Authors:** Yizhang Tong, Wei Wu, Wanjing Zhao, Yurui Xing, Hongti Zhang, Cheng Wang, Timothy B. Y. Chen, Anthony C. Y. Yuen, Bin Yu, Xianwu Cao, Xiaohong Yi

**Affiliations:** 1 Key Laboratory of Polymer Processing Engineering of Ministry of Education, Guangdong Provincial Key Laboratory of Technique and Equipment for Macromolecular Advanced Manufacturing, School of Mechanical and Automotive Engineering, South China University of Technology, Guangzhou 510640, China; 2 Jihua Laboratory, Foshan 528200, China; 3 School of Physical Science and Technology, Shanghai Tech University, Shanghai 201210, China; 4 School of Mechanical and Manufacturing Engineering, University of New South Wales, Sydney 2052, Australia; 5 State Key Laboratory of Fire Science, University of Science and Technology of China, Hefei 230026, China

**Keywords:** thermoplastic polyurethane, boron nitride, Co_3_O_4_, polyphosphazene, fire safety, mechanical property

## Abstract

Thermoplastic polyurethane (TPU) is widely used in daily life due to its characteristics of light weight, high impact strength, and compression resistance. However, TPU products are extremely flammable and will generate toxic fumes under fire attack, threatening human life and safety. In this article, a nanohybrid flame retardant was designed for the fire safety of TPU. Herein, Co_3_O_4_ was anchored on the surface of exfoliated ultra-thin boron nitride nanosheets (BNNO@Co_3_O_4_) via coprecipitation and subsequent calcination. Then, a polyphosphazene (PPZ) layer was coated onto BNNO@Co_3_O_4_ by high temperature polymerization to generate a nanohybrid flame retardant named BNNO@Co_3_O_4_@PPZ. The cone calorimeter results exhibited that the heat release and smoke production during TPU combustion were remarkably restrained after the incorporation of the nanohybrid flame retardant. Compared with pure TPU, the peak heat release rate (PHRR) decreased by 44.1%, the peak smoke production rate (PSPR) decreased by 51.2%, and the peak CO production rate (PCOPR) decreased by 72.5%. Based on the analysis of carbon residues after combustion, the significant improvement in fire resistance of TPU by BNNO@Co3O4@PPZ was attributed to the combination of quenching effect, catalytic carbonization effect, and barrier effect. In addition, the intrinsic mechanical properties of TPU were well maintained due to the existence of the PPZ organic layer.

## 1. Introduction

Thermoplastic polyurethane (TPU) elastomer is a type of polymer possessing superior toughness and excellent shock and corrosion resistance [1,2,3,4,5]. It has been utilized in wide-ranging applications in many areas, such as automotives, electronic appliances, daily necessities, etc. However, TPU is highly flammable and it is usually accompanied by a large amount of smoke and toxic gas release during its combustion, which limits the further applications of TPU and causes harm to the environment and human body [6,7,8,9,10]. Various types of functionalized 2D nanofillers have been incorporated into the TPU matrix to enhance its fire resistance. Yu et al. [11] utilized cetyltrimethyl ammonium bromide and tetrabutyl phosphine chloride to modify Ti_3_C_2_T_x_ ultra-thin nanosheets so as to achieve the remarkable dispersion of Ti_3_C_2_T_x_ in TPU. The functionalized Ti_3_C_2_T_x_ was able to greatly reduce the smoke product rate (SPR) and heat release rate (HRR) by more than 50%. Huang et al. [12] introduced phosphorus-containing HBPSi onto graphene oxide (GO) and mixed this novel type of flame retardant with TPU, which dramatically inhibited peak heat release rate (PHRR) and peak smoke product rate (PSPR) by 63.5 and 58.3% during combustion. The joint effects of the phosphorus radical scavenger, the barrier effect of GO, and the three-dimensional Si-O-Si framework of P-HBPSi played important roles in the improvement of the fire safety of TPU. Furthermore, MoS_2_ [13] and layered double hydroxide (LDH) were also utilized as effective flame retardants for TPU nanocomposites [14,15,16].

Recently, there has been a continuous interest in hexagonal boron nitride (h-BN) because of its unique features, such as high thermal stability, excellent chemical resistance, and superior thermal conductivity [17,18]. Thanks to its ultra-high temperature stability and unique 2D layered structure, BN can act as a barrier to inhibit the rapid heat and mass transfer of the polymer and delay the release of pyrolysis gas in the combustion of polymers [19,20,21]. Up to now, h-BN has been explored as a novel type of flame retardant filler for TPU [22,23,24,25,26,27]. Cai et al. [24] constructed a type of novel hybrid flame retardant for TPU based on SiO_2_, phytic acid (PA), and h-BN (h-BN@SiO_2_@PA). Compared with h-BN, the hybrid flame retardant h-BN@SiO_2_@PA overcame the chemical inertia of h-BN, achieving a better flame retardant effect and toxic gas suppression while greatly enhancing the mechanical properties of the TPU nanocomposite. Generally, the flame retardant efficiency of single flame retardants is not high, while the compounding of multiple types of flame retardants or constructing hybrid flame retardants can achieve high flame retardant efficiency and reduce the loading of the flame retardant as well as maintaining other properties of TPU [28,29,30,31]. However, the lip–lip interaction [32] between the B atom and N atom in adjacent layers of h-BN has a poor interfacial interaction with polymer matrices, which seriously affects the flame retardant efficiency of BN and deteriorates the mechanical properties of polymer nanocomposites. To overcome these shortcomings, it is necessary to functionalize the surface of the h-BN [33,34,35,36,37].

Polyphosphazene (PPZ) is a kind of polymer with an organic–inorganic hybrid structure, which can be prepared by the thermal ring opening polymerization or solution ring opening polymerization of Hexachlorocyclotriphosphazene (HCCP) [38]. Due to the existence of P and N elements and excellent thermal stability, PPZ has been utilized to improve the fire resistance of polymers [39,40,41,42]. Singh et al. [39] directly blended PPZ with TPU and its flame retardancy was evaluated by a limiting oxygen index (LOI) analyzer and UL-94 (vertical burning test), showing the rating of V-0 with an achieved LOI value of 31.4% for the TPU/PPZ blend. Qiu et al. [41] deposited PPZ on the surface of MoS_2_ nanosheets using thermal ring opening polymerization, which significantly reduced the PHRR and total heat release (THR) of EP. Furthermore, it has been proven that the combination of PPZ and transition metal elements can more effectively improve the fire resistance of flame retardants [43,44,45,46].

In this work, the exfoliated boron nitride nanosheets (BNNO) were decorated with Co_3_O_4_ nanoparticles and a PPZ layer to obtain a novel nanohybrid flame retardant. It was expected that the barrier effect of BNNO, combined with the promoting dispersion effect, the catalytic charring effect, and combustible gas dilution effect of Co_3_O_4_ and PPZ, was able to improve the flame retardant efficiency of the TPU composite. The effects of the flame retardant fillers on the morphology and mechanical performance of TPU nanocomposites were also investigated. Furthermore, the flame retardant mechanism of TPU/BNNO@Co_3_O_4_@PPZ nanocomposite was illustrated in detail.

## 2. Materials and Methods

### 2.1. Materials

Thermoplastic polyurethane (TPU, WHT-1570IC) with a density of 1.15 g/cm^3^ was purchased from Wanhua Chemical Group Co., Ltd. (Yantai, China). The hexagonal boron nitride (h-BN, purity > 99.5%) was obtained from Qingzhou Materials Co., Ltd. (Qingzhou, China). Cobalt (II) acetate tetrahydrate (C_4_H_6_CoO_4_·4H_2_O, 99.5%) was obtained from Shanghai Macklin Biochemical Co. Ltd., Shanghai, China. HCCP (purity, 98%) were supplied by J&K Chemical Co., Ltd. (Shanghai, China). Isopropanol (purity > 99.7%) and ammonia (NH_3_·H_2_O, 25 wt%) were supplied by Guangzhou Chemical Reagent Factory.

### 2.2. Preparation of BNNO@Co_3_O_4_@PPZ

**Figure 1:**
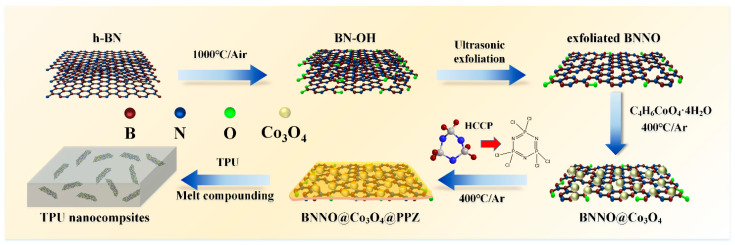
Preparation route of TPU/BNNO@Co_3_O_4_@PPZ nanocomposites.

The preparation routes of BNNO, BNNO@Co_3_O_4_, and BNNO@Co_3_O_4_@PPZ are illustrated in Figure 1. To achieve a well dispersion in isopropanol, h-BN was first treated at a high temperature to obtain hydroxyl groups on the h-BN surface. A certain amount of h-BN was put in a quartz boat and inserted into the center of a GSL-1100X-S tubular furnace (Hefei Kejing Materials Technology Co. Ltd., Hefei, China). Then the system temperature was raised to 1000 °C at 20 °C/min, followed by an isothermal process for 2 h in air atmosphere. The as-prepared oxidized BN was dispersed in 10 mL of isopropanol and then exfoliated with tip ultrasonication (25 kHz, 900 W) for 45 min. The resulting sample was denoted as BNNO.

The preparation method of Co_3_O_4_-decorated BNNO (BNNO@Co_3_O_4_) was based on previous studies [47]. First, 150 mg of BNNO was dispersed into 200 mL of DI water under tip sonication for 1 h. Then, 200 mg of C_4_H_6_CoO_4_·4H_2_O was mixed with 200 mL of DI water and transformed into the three-necked flask together with the dispersed BNNO dispersion. An amount of 7 mL of ammonia solution was added dropwise into the suspension. Then, the mixture was continuously stirred under condensation reflux for 4 h at 100 °C to ensure complete ion adsorption. Finally, the target BNNO@Co_3_O_4_ was obtained by thermal treatment at 400 °C in a muffle furnace.

The in situ synthesis of the polyphosphazene layer coated BNNO@Co_3_O_4_ was prepared via high temperature polymerization [41]. Typically, the BNNO@Co_3_O_4_ (2.5 g) was dispersed in isopropanol under ultrasonication for 1 h to form a homogeneous suspension. The 0.5 g of HCCP was added into the above solution by sonication. After that, the solution was stirred continuously at 70 °C to continue reacting for 2 h, and the mixture was dried at 90 °C to remove the isopropanol. The obtained solid was annealed in a tubular furnace at 700 °C for 3 h to synthesize a polyphosphazene layer. The resulting powder was centrifuged with isopropanol three times and abbreviated as BNNO@Co_3_O_4_@PPZ.

### 2.3. Preparation of TPU Nanocomposites

The TPU nanocomposites containing 2 wt% BNNO, BNNO@Co_3_O_4_, and BNNO@Co_3_O_4_@PPZ, respectively, were prepared by mixing TPU pellets with filler powder using a Brabendermixer at 170 °C for 10 min. Then the mixtures were made into different size specimens by hot-compression at 190 °C for various testing.

### 2.4. Characterization

The morphologies of the BNNO, BNNO@Co_3_O_4_, BNNO@Co_3_O_4_@PPZ, fracture surfaces of TPU nanocomposites, and micro-morphology of char residues were analyzed by a scanning electron microscope (SEM, FEI Quanta 250). The morphology and element distribution of fillers were measured on a transmission electron microscopy (TEM, JEOL JEM-2100F) equipped with an energy-dispersive X-ray spectrometer (EDX). The thickness of exfoliated BNNO was determined by atomic force microscopy (AFM, Veeco Multimode V) in the tapping mode. Fourier transform infrared spectroscopy (FT-IR) spectra were conducted on a FT-IR spectrometer (Nicolet iS50) in the wavenumber range from 400 to 2500 cm^−1^. X-ray diffraction (XRD) patterns were obtained from an X-ray diffractometer (AXS D2, Bruker, Germany) with Cu-Ka radiation. The elemental composition of fillers and char residue were analyzed by an X-ray photoelectron spectrometer (XPS, Kratos Axis Supra+) with a monochromatic Al Kα X-ray source (1486.6 eV) used for analysis. The thermal behavior of TPU nanocomposites was measured by a thermogravimetric analyzer (TGA, Netzsch TG 209 F1). The specimens of about 10 mg were heated from room temperature to 800 °C at a ramping rate of 10 °C/min under N_2_ atmosphere. The mechanical properties of TPU nanocomposites were measured by an electronic universal testing machine (Instron Model 5566). The dumbbell-shaped specimens were tested at a fixed speed of 200 mm/min. The reported values were the averages of five specimens. The real fire performance of TPU nanocomposites were measured on a cone calorimeter (i-CONE, Fire Testing Technology). The dimensions of the squared specimens was 100 × 100 × 3 mm^3^. The samples wrapped with aluminum foil were heated at a 35 kW/m^2^ external heat flux. The structure of the char residues after cone tests were tested on a RAMANLOG 6 laser Raman spectrometer equipped with a 532 nm laser. Thermogravimetric analyzer-Infrared spectroscopy (TG-IR) was executed with a METTLER TOLEDO TGA2 thermogravimetric analyzer coupled with a Thermofisher iS50 FTIR spectrophotometer under air atmosphere from 30 to 700 °C at a ramping rate of 20 °C/min.

## 3. Results

### 3.1. Characterization of BNNO@Co_3_O_4_@PPZ Hybrids

**Figure 2:**
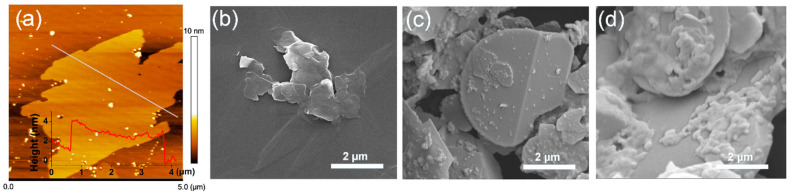
(**a**) AFM image (insert is thickness curve of the white line), SEM images of (**b**) BNNO, (**c**) BNNO@Co_3_O_4_, (**d**) BNNO@Co_3_O_4_@PPZ.

The microscopic morphology of exfoliated BNNO, BNNO@Co_3_O_4_, and BNNO@Co_3_O_4_@PPZ is shown in Figure 2. The AFM image in Figure 2a demonstrates that the BNNO with a 2–3 nm thickness is few-layer, indicating that the exfoliation of h-BN is successful [48]. In Figure 2b, the exfoliated BNNO exhibits a typical smooth lamellar structure with dimensions of around 5 μm. It should be noted that Co_3_O_4_ nanoparticles are found on the surface of BNNO@Co_3_O_4_, as shown in Figure 2c. After high-temperature polymerization, the PPZ layer has been deconjugated on the BNNO and covers the Co_3_O_4_ nanoparticles, which can be clearly seen in Figure 2d. The TEM image and EDX element mapping images in Figure S1 demonstrate that the existence of P and Co elements is uniformly distributed on the surface of BNNO@Co_3_O_4_@PPZ.

**Figure 3:**
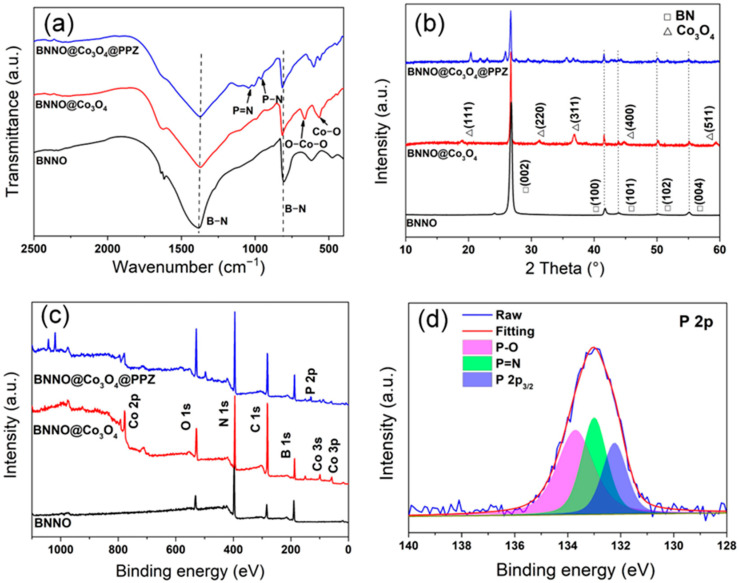
(**a**) FT-IR spectra; (**b**) XRD patterns; (**c**) XPS full survey of BNNO, BNNO@Co_3_O_4_, and BNNO@Co_3_O_4_@PPZ; and (**d**) the high resolution of P 2p of BNNO@Co_3_O_4_@PPZ.

The FT-IR spectra of exfoliated BNNO, BNNO@Co_3_O_4_, and BNNO@Co_3_O_4_@PPZ are shown in Figure 3a. In the spectrum of BNNO, the characteristic peaks bands of boron nitride at 1386 and 806 cm^-1^ can be observed, which are ascribed to the vibrations of the B-N bond. In the spectrum of BNNO@Co_3_O_4_, the absorption bands belong to the bridging vibration of O-Co-O and the stretching vibration of Co-O, which appear at 663 and 569 cm^−1^, respectively [49,50]. After the polymerization of PPZ, the strong bands at 940 and 1115 cm^−1^ are attributed to the stretching vibration of P-N and P = N bands in cyclotriphosphazene, indicating the existence of PPZ on the BNNO surface [41]. The XRD pattern of BNNO in Figure 3b shows multiple diffraction peaks at 26.8°, 41.8°, 43.5°, 50.2°, and 55.1°, corresponding to the BN crystal planes (002), (100), (101), (102), and (004), respectively [22]. After the decoration of Co_3_O_4_, several typical diffraction peaks at 19.0°, 31.2°, 36.5°, 44.8°, and 59.3° can be seen, which correspond to the (111), (220), (311), (400), and (511) planes of Co_3_O_4_. These characteristic peaks are well matched to the standard diffraction pattern of Co_3_O_4_ (JCPDS: 43-1003) [49]. This indicates that the Co_3_O_4_ is successfully decorated on the BNNO surface. In addition, there are some small peaks in the pattern of BNNO@Co_3_O_4_@PPZ, which originate from the amorphous PPZ. The intensity of the characteristic peaks of Co_3_O_4_ is reduced after coating the PPZ layer, which is consistent with other previous works [51,52]. Figure 3c shows the XPS full survey spectra of BNNO, BNNO@Co_3_O_4_, and BNNO@Co_3_O_4_@PPZ. Several intense peaks at 59, 99, and around 775–805 eV can be observed in the XPS spectrum of BNNO@Co_3_O_4_, which are ascribed to the Co 3p, Co 3s, and Co 2p peaks of Co_3_O_4_, indicating the occurrence of Co_3_O_4_ on the surface of BNNO [49]. To gain more insight, the XPS spectrum from 775 to 805 eV is enlarged in Figure S2, and the Co 2p exhibits two split peaks of Co 2p_3/2_ and Co 2p_1/2_, which appear at around 779 and 795 eV, respectively. A new peak of P 2p is clearly observed in the spectrum of BNNO@Co_3_O_4_@PPZ, confirming the successful coating of the PPZ layer on BNNO@Co_3_O_4_. In the high-resolution spectrum of P 2p peak in Figure 3d, the peak of P 2p can be fitted into three peaks, which are located at 132.2 eV (P 2p_3/2_ of P species), 133 eV (P = N bond), and 133.7 eV (P-O), respectively. As can be seen in Figure S3, BNNO, BNNO@Co_3_O_4_, and BNNO@Co_3_O_4_@PPZ have no obvious mass loss until 800 °C. Based on the above analysis, it can be reasonably proven that the BNNO@Co_3_O_4_@PPZ has been prepared successfully.

### 3.2. Fracture Surface Morphology of TPU Nanocomposites

**Figure 4:**
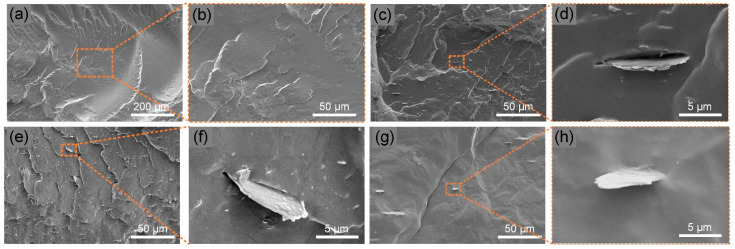
SEM images of the fractured surfaces of (**a**,**b**) TPU, (**c**,**d**) TPU/BNNO, (**e**,**f**) TPU/BNNO@Co_3_O_4_, and (**g**,**h**) TPU/BNNO@Co_3_O_4_@PPZ.

Figure 4 shows the SEM graphs of the brittle fracture surface of pure TPU, TPU/BNNO, TPU/BNNO@Co_3_O_4_, and TPU/BNNO@Co_3_O_4_@PPZ. In Figure 4a,b, pure TPU is brittle damaged with a smooth fracture surface. With the addition of BNNO and BNNO@Co_3_O_4_, the fracture surfaces displays an uneven condition, as shown in Figure 4c,e. Although there is no obvious agglomeration in the field of vision, as shown in Figure 4d,f, the debonding phenomenon occurs when observing the interface of the filler–matrix, indicating relatively poor compatibility [53]. In contrast, few fillers are exposed to the cross-sectional surface in the SEM image of the TPU/BNNO@Co_3_O_4_@PPZ in Figure 4g. In addition, a few BNNO@Co_3_O_4_@PPZ exposed on the surface are tightly bonded with the TPU matrix in Figure 4h. These results indicate that the introduction of a PPZ layer can improve the interfacial interaction of the filler–matrix.

### 3.3. Thermal Properties of TPU Nanocomposites

**Figure 5:**
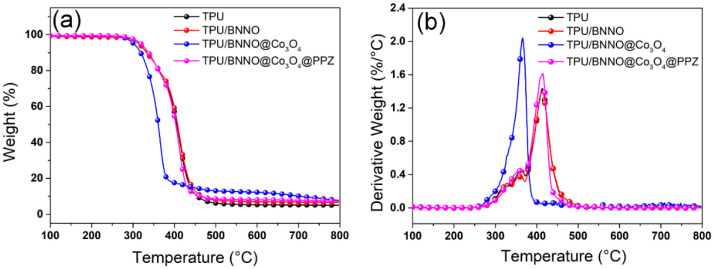
Thermal stability of TPU nanocomposites, (**a**) TGA curves, and (**b**) DTG curves at N_2_ atmosphere.

The effects of different fillers on the pyrolysis behavior of TPU and its nanocomposites were tested by TG under nitrogen atmosphere, and the curves and data are displayed in Figure 5 and Table 1. The temperature at 5 wt% weight loss is defined as Temp_d5%_, while the temperature at maximum mass loss rate is named as Temp_max_. According to the TGA curves in Figure 5a, except for TPU/BNNO@Co_3_O_4_, TPU/BNNO, and TPU/BNNO@Co_3_O_4_@PPZ, both suffer from the similar two-stage decomposition of TPU. In the first stage of decomposition, the degradation of the urethane bond in TPU produces diisocyanate, glycol, and carbon dioxide, while the second step is related to the thermal decomposition of the polyol segment in TPU soft segment [6]. As shown in Table 1, the introduction of Co_3_O_4_ greatly changed the pyrolysis behavior of TPU. The Temp_d5%_value of TPU/BNNO@Co_3_O_4_ drops by 12.4 °C to 301.6 °C. According to Figure 5b, the addition of Co_3_O_4_ induces TPU degradation to a one-stage process, which is due to the catalytic degradation of TPU by transition metals, thus resulting in the early decomposition of the second stage [54]. However, the TPU/BNNO@Co_3_O_4_@PPZ showed the highest thermal decomposition temperature among other nanocomposites, and its Temp_d5%_ is 4.7 °C higher than TPU, which is attributed to the superior thermal stability and catalytic charring effect of PPZ. In addition, the char residue at 800 °C of TPU/BNNO@Co_3_O_4_ and TPU/BNNO@Co_3_O_4_@PPZ is higher than TPU. Due to the catalytic carbonization of PPZ and Co_3_O_4_, the char residue of TPU nanocomposites was increased to 7.8% and 7.42%, respectively. The formation of char residue can serve as a physical barrier that helps to inhibit the release of smoke and the transfer of mass and heat during the combustion, thus helping to improve the fire safety of TPU.

### 3.4. Fire Safety of TPU Nanocomposites

**Figure 6:**
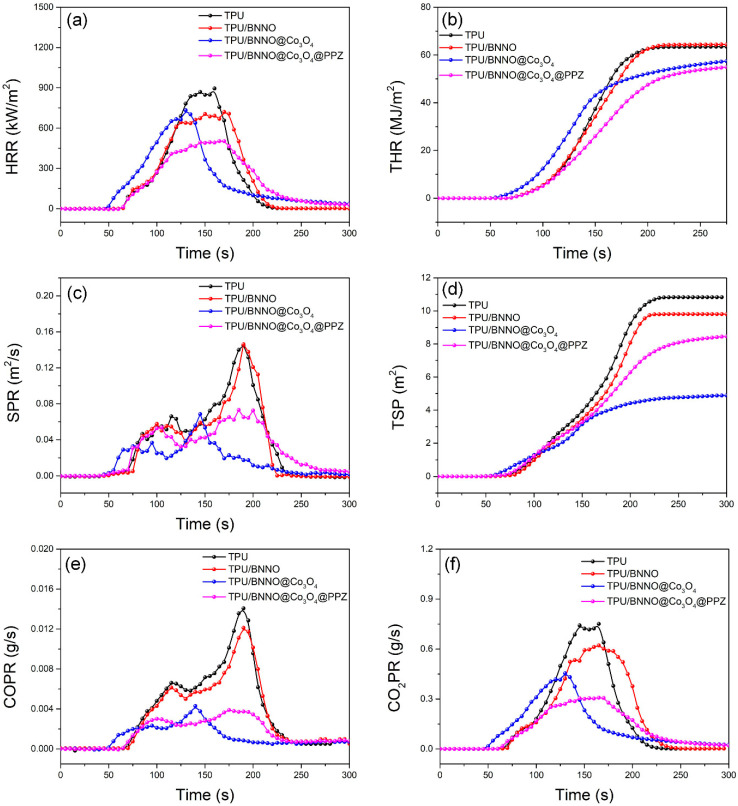
(**a**) HRR, (**b**) THR, (**c**) SPR, (**d**) TSR, (**e**) COPR, and (**f**) CO_2_PR as a function of time of TPU and its nanocomposites obtained from the cone calorimeter.

Cone calorimetry can simulate the combustion state in the real environment and monitor the heat release and gas release during the combustion, which is a powerful means for assessing the fire performance of materials (Figure 6 and Table 2) [55]. In Figure 6a,b, it can be seen that an obvious suppression in PHRR and THR with the incorporation of BNNO@Co_3_O_4_ and BNNO@Co_3_O_4_@PPZ nanohybrid flame retardants. The BNNO@Co_3_O_4_@PPZ possesses the highest flame retardancy efficiency among all samples under the same filler content. With 2 wt% BNNO@Co_3_O_4_@PPZ, the PHRR and THR value of nanocomposites are reduced to 503.1 kW/m^2^ and 56.9 MJ/m^2^, respectively, which is 44.1% and 10.3% lower than that of pure TPU (900.8 kW/m^2^ and 63.46 MJ/m^2^). In Table 2, the TTI of pure TPU is 72 s, and with the appearance of Co_3_O_4_, the TTI of TPU/BNNO@Co_3_O_4_ and TPU/BNNO@Co_3_O_4_@PPZ reduced to 53 s and 68 s, respectively. This is ascribed to the degradation of TPU being accelerated due to catalytic degradation of Co_3_O_4_, which is consistent with TGA results [56,57].

Generally speaking, the spread of smoke is the biggest obstacle for humans escaping from a fire environment and the carbon monoxide (CO) from incomplete combustion directly threatens human life. Hence, the suppression effect on smoke is also a crucial index for assessing the performance of flame retardants. From Figure 6, the curves indicate that pure TPU produces huge amounts of smoke during combustion, with a PSPR of 0.1497 m^2^/s and a high TSP value of 10.8 m^2^. When BNNO is added, the PSPR and TSP of TPU/BNNO slightly decrease to 0.1462 m^2^/s and 9.8 m^2^, respectively. With 2 wt% BNNO@Co_3_O_4_ and BNNO@Co_3_O_4_@PPZ, the values of PSPR were decreased by 54.2% and 51.2%, respectively, exhibiting the superior efficiency of the nanohybrid flame retardants on smoke suppression. As for the release of CO, the PCOPR of TPU/BNNO@Co_3_O_4_ and TPU/BNNO@Co_3_O_4_@PPZ decrease from 69.7% and 72.5% to 0.0043 and 0.0039 g/s, respectively, much less than those to that of pure TPU (0.0142 g/s). Moreover, the peak values of carbon dioxide production (PCO_2_PR) for TPU nanocomposites show a similar decline tendency as PCOPR. Compared to pure TPU, TPU/BNNO@Co_3_O_4_ and TPU/BNNO@Co_3_O_4_@PPZ show a 40.1 and 59.3% reduction in the PCO_2_PR, respectively. The results of cone calorimetry show that the introduction of Co_3_O_4_ can effectively suppress toxic smoke production, especially for CO, during TPU combustion. The presence of PPZ in the hybrid flame retardant additive can further reduce the heat release and significantly enhance the fire performance due to the phosphorus element in PPZ [14,26,58].

### 3.5. Analysis of Char Residues after Combustion

**Figure 7:**
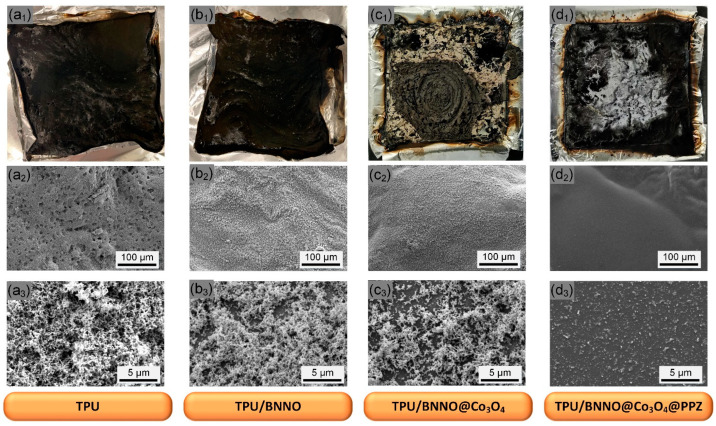
Digital photos of the char residues and SEM images (low magnification and high magnification) pure TPU (**a_1_**–**a_3_**), TPU/BNNO (**b_1_**–**b_3_**), TPU/BNNO@Co_3_O_4_ (**c_1_**–**c_3_**), and TPU/BNNO@Co_3_O_4_@PPZ (**d_1_**–**d_3_**).

To deeply explore the mechanism of the nanohybrid flame retardant, the digital photos and micromorphology of the char residues after the cone calorimeter test are shown in Figure 7. From the digital photos, loose and brittle residual char can be observed in pure TPU, and plentiful open holes are distributed on the surface. In contrast, the char formation of TPU nanocomposites containing flame retardants gradually becomes continuous and dense. According to the SEM images corresponding to the digital photos, for the pure TPU, the surface of the char residue is still honeycombed and densely covered with a large number of tiny holes on the microscopic scale, which provides access for flammable gases and toxic volatiles in and out during the combustion process, which is very unfavorable to fire safety. The introduction of BNNO and Co_3_O_4_ reduced the number of micropores, but the soft honeycomb-like structure can still be observed under high magnification. However, the char residue of TPU nanocomposites loaded with BNNO@Co_3_O_4_@PPZ nanohybrid flame retardant shows a compact and continuous structure, and there are tiny obvious holes in the field of vision. The formation of a dense char structure is ascribed to the catalytic charring effect of Co_3_O_4_ and PPZ during the combustion, which is conducive to retarding the mass and heat transfer and escape of pyrolysis volatiles, thus improving the flame retardancy of the TPU [59].

**Figure 8:**
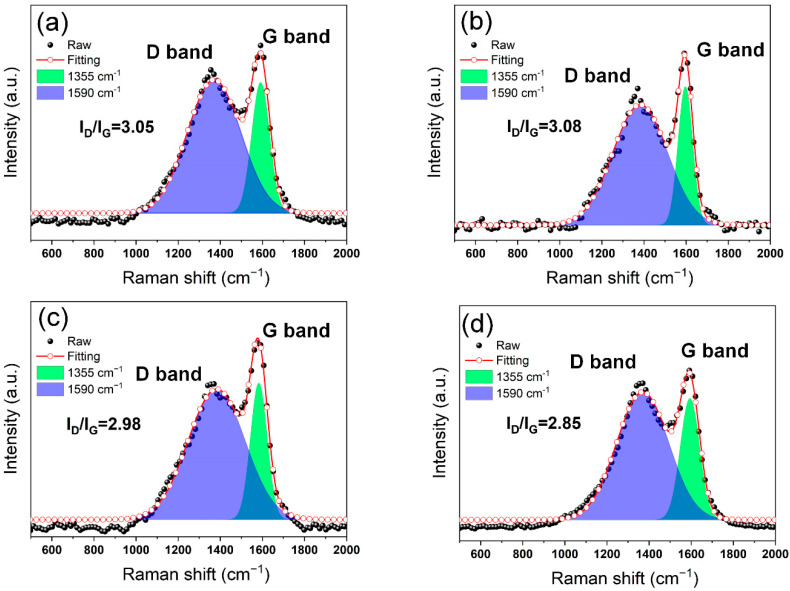
Raman spectra of char residue of (**a**) TPU, (**b**) TPU/BNNO, (**c**) TPU/BNNO@Co_3_O_4_, and (**d**) TPU/BNNO@Co_3_O_4_@PPZ.

**Figure 9:**
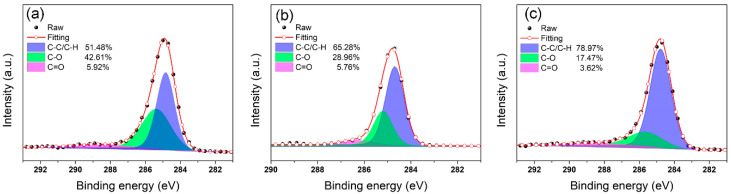
The high resolution of C1s XPS spectra of char residue of (**a**) pure TPU, (**b**) TPU/BNNO@Co_3_O_4_, and (c) TPU/BNNO@Co_3_O_4_@PPZ.

Generally speaking, the char-forming quality is positively correlated with its graphitization degree. The integrated area ratio of D-band and G-band (I_D_/I_G_) in Raman spectra is often used to reflect the graphitization degree of samples. The lower I_D_/I_G_ means the higher graphitization degree and quality of the char layer [7,60]. In Figure 8, it is noteworthy that the values for I_D_/I_G_ of pure TPU, TPU/BNNO, TPU/BNNO@Co_3_O_4_, and TPU/BNNO@Co_3_O_4_@PPZ are 3.06, 3.08, 2.98, and 2.85, respectively, which indicates the highest graphitization degree for the char residue of TPU/BNNO@Co_3_O_4_@PPZ. Furthermore, through the peak splitting of the high-resolution C1s XPS spectra, the concentration of atomic bond types of residual char can be quantitatively obtained to further assess the degree of graphitization. From Figure 9, the percentage of C-C bonds peak area of pure TPU, TPU/BNNO@Co_3_O_4_, and TPU/BNNO@Co_3_O_4_@PPZ are calculated by XPS analysis software AVANTAGE to be 51.48%, 65.28%, and 78.97%, respectively, which means the decreasing concentration of C-O, C=O, and oxidation degree. This result further demonstrates the higher graphitization degree of TPU/BNNO@Co_3_O_4_@PPZ.

### 3.6. Vapor-Phase Analysis

**Figure 10:**
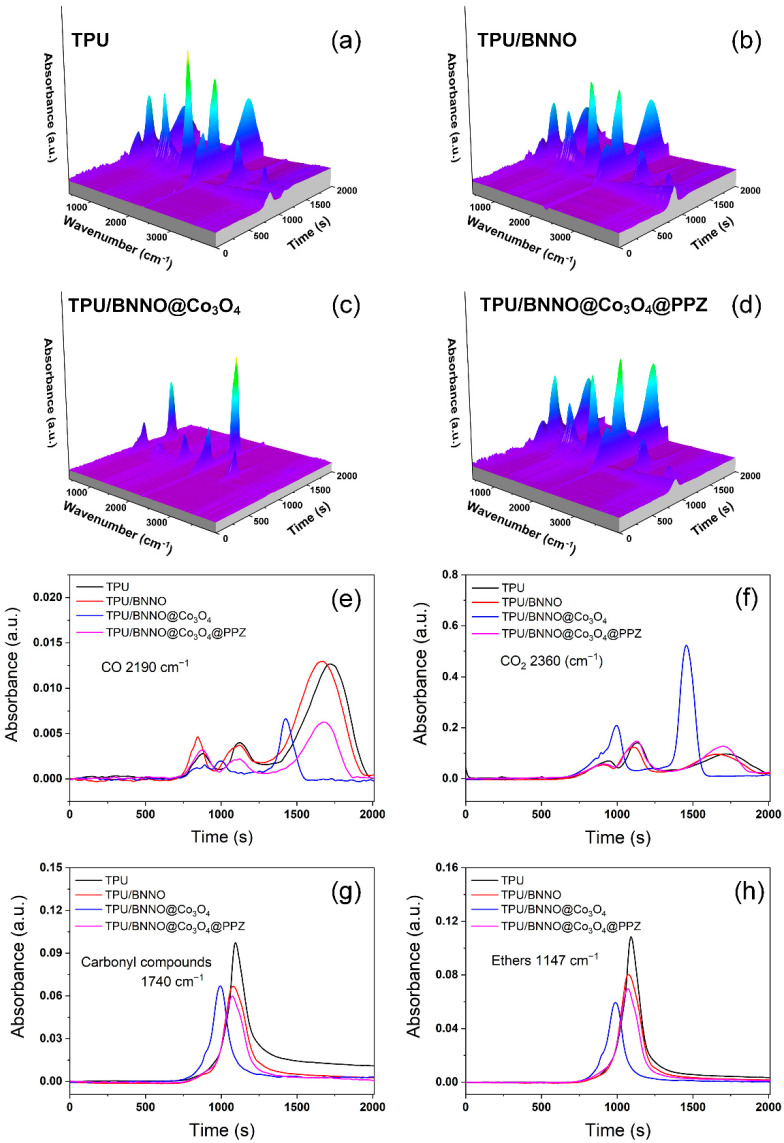
3D TG-FTIR spectra of (**a**) TPU, (**b**) TPU/BNNO, (**c**) TPU/BNNO@Co_3_O_4_, and (**d**) TPU/BNNO@Co_3_O_4_@PPZ. The absorbance of pyrolysis products for TPU, TPU/BNNO, TPU/BNNO@Co_3_O_4_, and TPU/BNNO@Co_3_O_4_@PPZ as a function of time: (**e**) CO, (**f**) CO_2_, (**g**) carbonyl compounds, and (**h**) ethers.

The TG-IR technique was used to simulate the types and intensities of gases released during the pyrolysis of TPU and its nanocomposites under air atmosphere to further reveal the mechanism of the flame retardant improvement of TPU by BNNO@Co_3_O_4_ and BNNO@Co_3_O_4_@PPZ. The 3D TG-IR spectra depicted in Figure 10a–d demonstrate that the addition of Co_3_O_4_ significantly increases the intensity of gas volatilization, whereas the introduction of PPZ can suppress the release of volatile gaseous. In fact, most of the gas volatilization of TPU/BNNO@Co_3_O_4_ comes from the massive production of carbon dioxide (CO_2_), which is of great significance for the production of non-combustible gases in flame retardant applications. Therefore, the release intensity and time of various gases must be analyzed in detail. According to Figure 10e, the intensity of CO released by TPU/BNNO@Co_3_O_4_ is the lowest at around 750–1250 s, and there is a strong but short period of CO release at around 1250 s. Meanwhile, the other three materials release a certain amount of CO in the time period of 750–1250 s, and a large amount of CO is released for a long time after 1500 s. Based on the CO_2_ release curves in Figure, it can be concluded that the catalytic effect of transition metals in Co_3_O_4_ on decomposition makes CO, which is produced by incomplete combustion, and forming CO_2_ when completely burned. The presence of the PPZ layer can suppress the release of CO due to its catalytic effect. However, the release of CO_2_ is greatly weakened. This may be due to the coating of PPZ with strong thermal stability, and the catalytic effect of Co_3_O_4_ has not been fully exerted. In the real combustion process, PPZ is totally pyrolyzed at a higher temperature. Meanwhile, the flame retardant effects of Co_3_O_4_ and PPZ can be exerted at the same time. In addition, the release of carbonyl compounds and ethers is greatly suppressed during the combustion of TPU/BNNO@Co_3_O_4_@PPZ in Figure 10g,h, which is also due to the more complete combustion under the catalysis of Co_3_O_4_ and PPZ. The results of TG-FTIR can reasonably explain that BNNO shows the optimal inhibition effect on the release of gas degradation products of BNNO@Co_3_O_4_@PPZ due to the synergistic effects of Co_3_O_4_ and PPZ.

### 3.7. Flame Retardant Mechanism of TPU Nanocomposites

**Figure 11:**
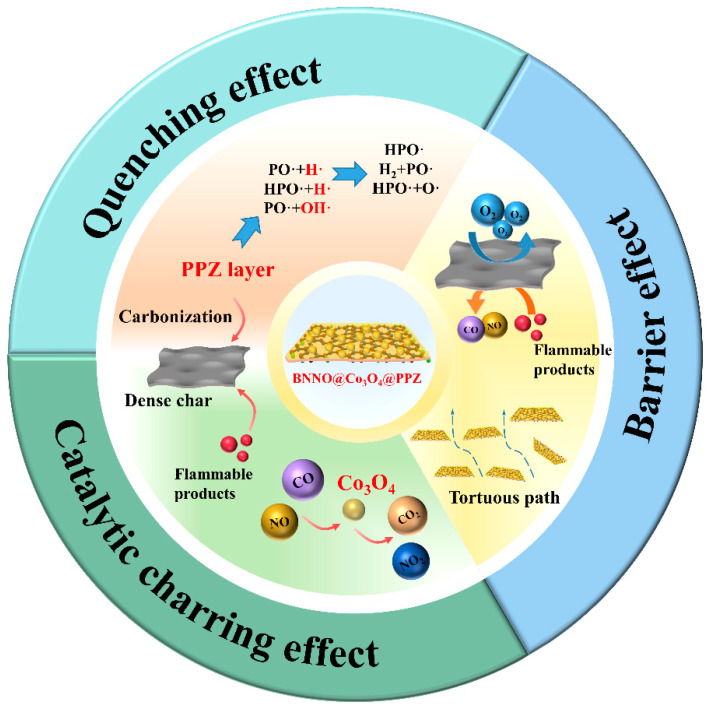
Diagram of flame-retardant mechanisms for TPU nanocomposites.

Based on the analysis of the gaseous and condensed phases, the excellent flame retardancy of BNNO@Co_3_O_4_@PPZ nanohybrid flame retardant, as shown in Figure 11, can be ascribed to the joint effect of BNNO, Co_3_O_4_, and PPZ. During the combustion, Co_3_O_4_ in the inner layer of nanohybrid flame retardant can catalyze CO, NO, and other combustible pyrolysis gases to generate CO_2_, NO_2_, and non-combustible gases, thus suppressing the toxic gases [41,56,61,62], which is consistent with the results of TG-IR. PPZ and Co_3_O_4_ can catalyze the formation of carbon, which is conducive to the formation of a denser char layer. The final dense char residue structure further restricted the permeation of oxygen and flammable products and acted as a barrier to heat. Meanwhile, the radicals, including •P, •PO, and •HPO generated during the pyrolysis of PPZ can combine with inflammable •H and •OH to remove flammable free radicals. This quenching effect would cut off the combustion and thus significantly reduce the heat release [12,43,63]. The “tortuous path” caused by the 2D layered structure of BNNO can also delay the transfer of combustible pyrolysis gas to the fire area to some extent. In addition, as a carrier, BNNO makes Co_3_O_4_ and PPZ disperse more uniformly and also enables toxic smoke to fully react with Co_3_O_4_ during escape.

To highlight the significant improvement of BNNO@Co_3_O_4_@PPZ in the retardancy of TPU nanocomposites, a comprehensive comparison of many approaches devoted to suppressing the heat and toxic gas release during the TPU combustion, including this work, is given in Table 3. It can be seen that the direct introduction of cobalt-containing nanofillers [64] or the loading of cobalt on two-dimensional nanofillers [65] can only achieve the simultaneous suppression of heat release and smoke release with difficulty, and the thermal stability of the nanocomposite is deteriorated, which is not practical for specific applications. In this work, we hybridize cobalt (Co_3_O_4_) and phosphorus-containing polymers (PPZ) with BNNO. The introduction of BNNO@Co_3_O_4_@PPZ to TPU leads to remarkable reductions in PHRR (44.1%), PSPR (51.2%), PCOPR (72.5%), and PCO_2_PR (59.3%), achieving simultaneous suppression of heat release and flue gas release, which are better than most reported results. Importantly, the retardancy of TPU is improved without deterioration of the thermal properties and mechanical properties.

### 3.8. Mechanical Properties of TPU Nanocomposites

**Figure 12:**
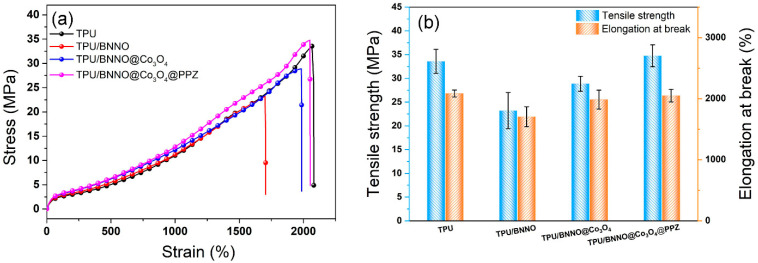
(**a**) Strain–stress curves, (**b**) tensile properties of TPU nanocomposites.

The addition of flame retardant does not affect the excellent mechanical properties of TPU and can even be used as a reinforcing agent, which is the most ideal case. In Figure 12, good strength and toughness with a tensile strength of 33.58 MPa and an elongation at break of 2085% was obtained in pure TPU, while the existence of BNNO leads to a sharp decrease in the tensile properties of TPU/BNNO. The poor interfacial interaction, as shown in Figure 4c,d, between BNNO and TPU makes the sample easier to break under tensile stress. After the decoration of Co_3_O_4_, the tensile performance of TPU/BNNO@Co_3_O_4_ was enhanced compared to TPU/BNNO. It can be attributed that the introduction of Co_3_O_4_ nanoparticles can hinder the agglomeration of BNNO [65]. TPU/BNNO@Co_3_O_4_@PPZ obtained the highest tensile strength (34.77 MPa) and good elongation at break (2055%), basically maintaining the original mechanical properties of TPU. This is mainly because the organic PPZ layer improves the interfacial adhesion between the filler–matrix, thus realizing the enhancement of mechanical properties of TPU@Co_3_O_4_@PPZ [41].

## 4. Conclusions

In this work, the transition metal oxide Co_3_O_4_ was decorated onto the surface of oxidized h-BN (BNNO@Co_3_O_4_). Then the PPZ was coated on the surface of BNNO@Co_3_O_4_ (BNNO@Co_3_O_4_@PPZ) via the high-temperature polymerization of HCCP. With the content of 2 wt% BNNO@Co_3_O_4_@PPZ, the PHRR and THR of the TPU/BNNO@Co_3_O_4_@PPZ nanocomposite were significantly reduced by 44.1% and 10.4%, respectively, as compared with those of pure TPU. Moreover, BNNO@Co_3_O_4_@PPZ also exhibited a remarkable suppression of smoke production. The PSPR, the PCOR, and the PCO_2_R of TPU/BNNO@Co_3_O_4_@PPZ nanocomposites had a substantial decline of 51.2, 72.5, and 59.4%, respectively. After analyzing the gaseous and condensed phases of combustion products of nanocomposites, the main mechanisms for the nanohybrid flame retardant BNNO@Co_3_O_4_@PPZ to enhance the safety performance of TPU during combustion were ascribed to the quenching effect of pyrolytic products of PPZ on flammable-free radicals and the barrier effect of the dense and continuous graphitized char layer formed by the catalytic charring effect of PPZ and Co_3_O_4_ on heat and gas delivery. Moreover, SEM graphs showed that BNNO@Co_3_O_4_@PPZ was uniformly dispersed in the TPU matrix due to the coating of PPZ and exhibited a close interface bonding between filler and polymer matrix. Thus, the BNNO@Co_3_O_4_@PPZ was able to improve the fire safety of TPU while maintaining its original mechanical properties. This work provides a simple and effective method and structure of a hybrid flame retardant for improving the flame retardancy and smoke suppression of TPU during ignition.

## Figures and Tables

**Table 1:** TGA data of TPU, TPU/BNNO, TPU/BNNO@Co_3_O_4_, and TPU/BNNO@Co_3_O_4_@PPZ nanocomposites.

Samples	Temp_d5%_ (°C)	Temp_max_ (°C)	R_800°C_ (wt%)
TPU	314.0	413.6	5.08
TPU/BNNO	314.2	413.8	6.81
TPU/BNNO@Co_3_O_4_	301.6	365.8	7.8
TPU/BNNO@Co_3_O_4_@PPZ	318.7	414.5	7.42

**Table 2:** Relevant data of TPU nanocomposites by cone calorimeter test.

Samples	TTI(s)	PHRR (kW/m^2^)	THR (MJ/m^2^)	PSPR (m^2^/s)	TSP (m^2^)	PCOPR (g/s)	PCO_2_PR (g/s)
Pure TPU	72	900.8	63.5	0.1497	10.8	0.0142	0.758
TPU/BNNO	72	724.7	64.5	0.1462	9.8	0.0123	0.663
TPU/BNNO@Co_3_O_4_	53	732.9	60.0	0.0685	4.9	0.0043	0.454
TPU/BNNO@Co_3_O_4_@PPZ	68	503.1	56.9	0.073	8.6	0.0039	0.308

**Table 3:** Comparisons in heat and toxic gases release of TPU nanocomposites reported in prior work and this work.

Sample	PHRR	PSPR	PCOPR	PCO_2_PR
BN@P-PEI [22]	−34.4%	−26.8%	-	-
h-BN@SiO2@PA [24]	−23.5%	−29.2%	−26.8%	−11.0%
h-BN-PPy-PA-Cu2+ [25]	−35.6%	−31.8%	-	-
BNO/PANI [27]	−32.6%	+9.1%	0%	−32.2%
Co_3_O_4_-Tannic acid [56]	−18.9%	−33.3%	−11.4%	−10.5%
GO-DOPO [60]	−35.8%	−50%	−57.1%	−36.5%
Black phosphorus-HPL [64]	−49.9%	−45.8%	−37.5%	−32.5%
Co_3_O_4_/GNS [65]	−(<)10%	-	−18.2%	-
Co(OH)2 [66]	−38.7%	−33.3%	−81.0%	-
BNNO@Co_3_O_4_@PPZ (This work)	−44.1%	−51.2%	−72.5%	−59.3%

## Data Availability

The raw/processed data generated in this work are available upon request from the corresponding author.

## Supplementary Materials

The following supporting information can be downloaded at: https://www.mdpi.com/article/10.3390/polym14204341/s1, Figure S1: (a) TEM image, (b) EDX elemental mapping of B, N, P, and Co of BNNO@Co_3_O_4_@PPZ. Figure S2: High resolution of Co 2p of BNNO@Co_3_O_4_@PPZ; Figure S3: TGA curves of BNNO, BNNO@Co_3_O_4_, and BNNO@Co_3_O_4_@PPZ.
