# Supplementary material for: Nanohybrid of Co3O4 Nanoparticles and Polyphosphazene-Decorated Ultra-Thin Boron Nitride Nanosheets for Simultaneous Enhancement in Fire Safety and Smoke Suppression of Thermoplastic Polyurethane
Source: Polymers (Basel). 2022 Oct 15;14(20):4341. doi: 10.3390/polym14204341 (PMC9606935; doi:10.3390/polym14204341)
Supplement: Supplementary file 1 [file polymers-14-04341-s001.zip › polymers-1966041-supplementary.pdf]

polymers

Supplementary Material

Nanohybrid of Co_3O_4 Nanoparticles and Polyphosphazene Decorated Ultra-Thin Boron Nitride Nanosheets for Simultaneous Enhancement in Fire Safety and Smoke Suppression of Thermoplastic Polyurethane

Yizhang Tong [1,†], Wei Wu [2,†], Wanjing Zhao [1], Yurui Xing [3], Hongti Zhang [3], Cheng Wang [4], Timothy B. Y. Chen [4], Anthony C. Y. Yuen [4], Bin Yu [5], Xianwu Cao [1,*] and Xiaohong Yi [2,*]

[1] Key Laboratory of Polymer Processing Engineering of Ministry of Education, Guangdong Provincial Key Laboratory of Technique and Equipment for Macromolecular Advanced Manufacturing, School of Mechanical and Automotive Engineering, South China University of Technology, Guangzhou 510640, China
[2] Jihua Laboratory, Foshan 528200, China
[3] School of Physical Science and Technology, Shanghai Tech University, Shanghai 201210, China
[4] School of Mechanical and Manufacturing Engineering, University of New South Wales, Sydney 2052, Australia
[5] State Key Laboratory of Fire Science, University of Science and Technology of China, Hefei 230026, China
* Correspondence: ppeme@scut.edu.cn (X.C.); yixh@jihualab.ac.cn (X.Y.)
† The authors contribute equally to this work.

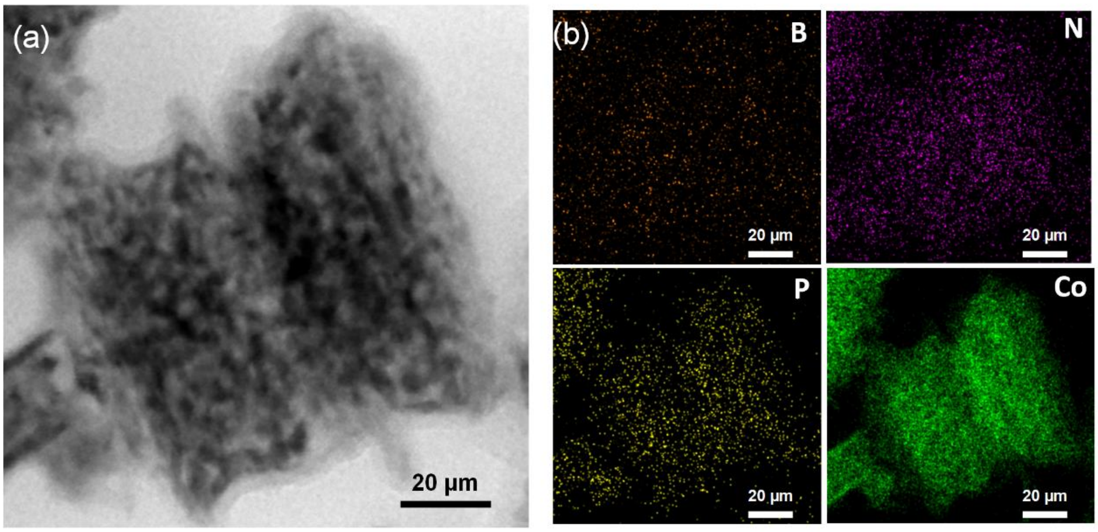

Figure S1. (a) TEM image, (b) EDX elemental mapping of B, N, P, and Co of BNNO@Co_3O_4@PPZ.

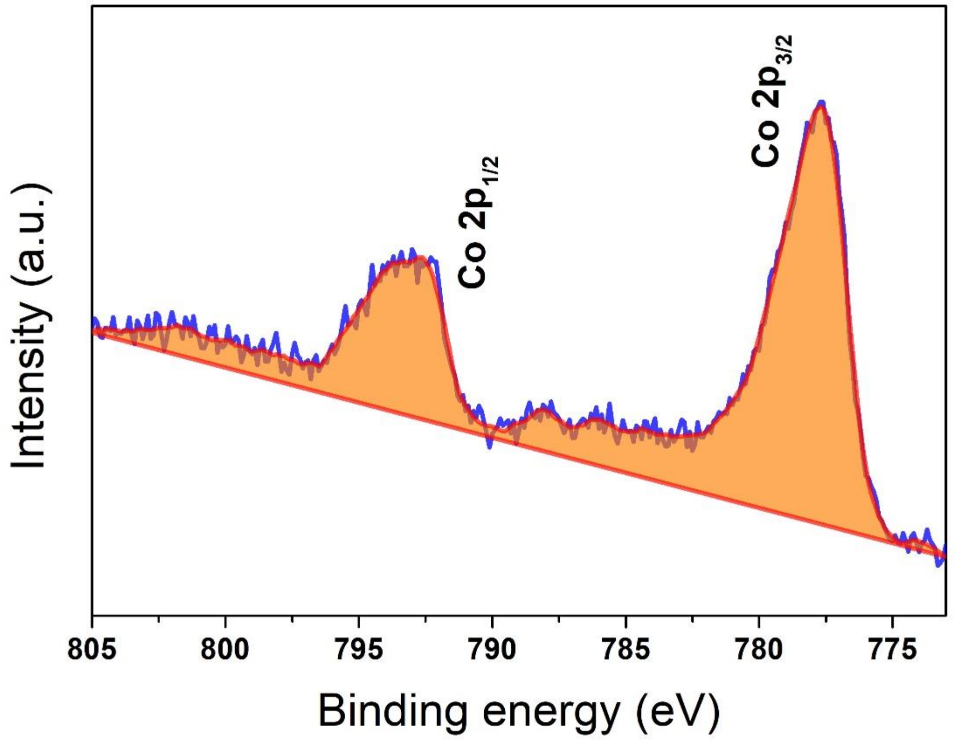

Figure S2. High resolution of Co 2p of BNNO@Co_3O_4@PPZ.

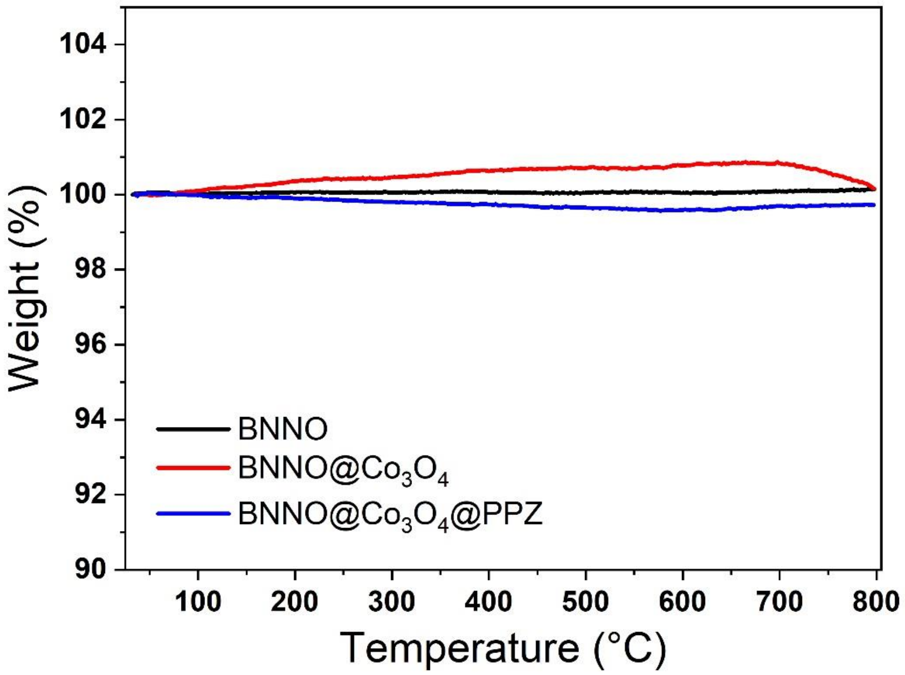

Figure S3. TGA curves of BNNO, BNNO@Co_3O_4, and BNNO@Co_3O_4@PPZ.
